# Current views of drought research: experimental methods, adaptation mechanisms and regulatory strategies

**DOI:** 10.3389/fpls.2024.1371895

**Published:** 2024-04-04

**Authors:** Xiyue Wang, Xiaomei Li, Wei Zhao, Xiaomin Hou, Shoukun Dong

**Affiliations:** ^1^ College of Agriculture, Northeast Agricultural University, Heilongjiang, Harbin, China; ^2^ College of Agriculture, Heilongjiang Agricultural Engineering Vocational College, Heilongjiang, Harbin, China; ^3^ Millet Research Institute, Qiqihar Sub-Academy of Heilongjiang Academy of Agricultural Sciences, Heilongjiang, Qiqihar, China

**Keywords:** drought stress, physiological response, molecular mechanism, drought-resistant strategies, experimental methods

## Abstract

Drought stress is one of the most important abiotic stresses which causes many yield losses every year. This paper presents a comprehensive review of recent advances in international drought research. First, the main types of drought stress and the commonly used drought stress methods in the current experiment were introduced, and the advantages and disadvantages of each method were evaluated. Second, the response of plants to drought stress was reviewed from the aspects of morphology, physiology, biochemistry and molecular progression. Then, the potential methods to improve drought resistance and recent emerging technologies were introduced. Finally, the current research dilemma and future development direction were summarized. In summary, this review provides insights into drought stress research from different perspectives and provides a theoretical reference for scholars engaged in and about to engage in drought research.

## 1 Introduction

There are many types of drought. The World Meteorological Organization has defined six types of drought: (1) Meteorological drought: caused by insufficient precipitation, it is indicated as the absolute value of a specified length of precipitation; (2) Climate drought: caused by insufficient precipitation, not by a specific number, it is represented by the ratio of the average or normal value; (3) Atmospheric drought, which is influenced by temperature, humidity, wind speed, air pressure, and other meteorological variables in addition to precipitation; (4) Drought in agriculture, which is mostly associated with soil moisture content, plant ecology, and maybe the behavior of a specific crop; (5) Drought in hydrology, which is primarily associated with a decline in river flow, a reduction in lake or reservoir capacity, and a decline in groundwater level; (6) Water management drought, which is defined as a lack of water resulting from either the actual usage of water management or the devastation of infrastructure (WMO, 1975). At present, we often say that drought usually refers to agricultural drought and meteorological drought. For example, Heilongjiang Province is a concentrated soybean producing area in China, and it is one of the areas with the highest frequency of spring drought. The frequency of spring drought is as high as about 70% (Zhu et al., 2020), which often refers to meteorological drought. The occurrence of agricultural drought is a complex process. The term “agricultural drought” describes the situation when a lack of water in crops due to outside environmental causes interferes with their regular growth and development, resulting in lower yields or a loss of harvest (Zhang et al., 2021a); in academic research, it is usually based on the specific research of agricultural drought. In crop production, due to different environmental conditions often suffer biotic or abiotic stress, these factors will cause a certain loss of yield, is the main factor affecting crop production (Feng et al., 2020); among these abiotic stresses, drought is one of the main limiting factors for crop production, and the loss of crop yield caused by drought alone exceeds the sum of all pathogens (Gupta et al., 2020). Therefore, improving the drought resistance of crops has been a research hotspot for many years.

## 2 Drought research methods

To better study the response of plants to drought stress, researchers have developed a variety of different methods to simulate drought stress. In this section, the current drought research methods and their advantages and disadvantages are further summarized.

**Figure 1:**
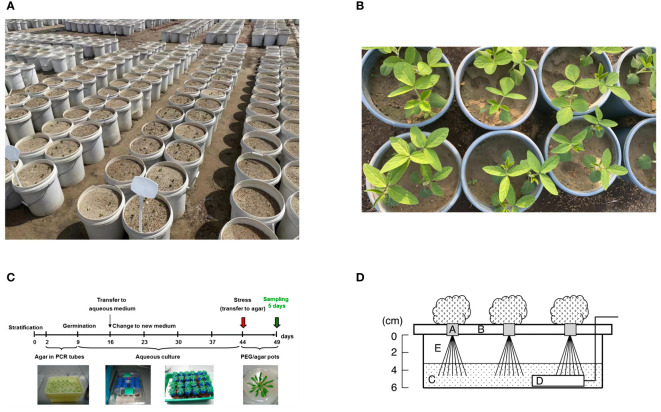
Experimental drought models. **(A)**, soil drought pot experiment based on water control method; **(B)**, drought experiment based on sand culture + PEG; **(C)**, agar-based PEG infusion Arabidopsis thaliana model, established by overlaying solidified agar medium with PEG8000 solution for five days (Osmolovskaya et al., 2018). **(D)**, Schematic view of the hydroponic system used to induce drought stress to roots of leafy vegetables. Five-day-old seedlings in growing medium cubes **(A)** were transferred to a hydroponic system. The seedlings were supported by a plastic foam bed **(B)** and irrigated with a nutrient solution **(C)** that was aerated with an air stone **(D)**. After 23 d, seedlings were exposed to stress treatment by lowering the water level from 0 to 4 cm **(E)** in the solution tub for various periods before harvesting. Conditions for growth and treatments were as follows: 20°C (68.0°F), 14-h light/10-h dark photoperiod with light provided by cool-white fluorescent lamps (photosynthetic photon flux of 60/140 μmol·m^−2^·s^−1^ for germination/cultivation) (Koyama et al., 2012).

The first is the soil pot water control method, which is also one of the main drought simulation methods (**Figure 1A**). Researchers usually use weighing method or soil moisture meter to control the soil moisture content (Du et al., 2020; Dong et al., 2023). In the study of Dong et al. (2023), the GB/T 32136-2015 (China national standard) classification method was used to determine the drought level, that is, typical irrigation involves a soil relative water content of 65-75%; mild drought, moderate drought, and severe drought, corresponding to a soil relative water content of 50-60%, 40-50%, and 30-40%, respectively. An obvious advantage of this method is that it is very close to the natural environment and the situation in field production, which is a process of progressive water loss. The research based on this method can basically show the real state of the plant which is close to the nature. However, the disadvantages of this method are also obvious. The biggest problem is the water potential (ψw); A physical measurement of the condition of free energy in water is called water potential. It can accurately characterize the water state of plants when used in conjunction with associated physiological data and is appropriate for a variety of soil types and environmental circumstances. By providing a fundamental description of plant water status, ψw fosters data integration and exchange across research disciplines and deepens our understanding of trials linked to drought (Juenger and Verslues, 2023). However, soil drought usually controls the soil moisture content within a certain range, which also leads to the fact that ψw is always in a dynamic change, which greatly reduces the repeatability of the experiment and is affected by many factors, including soil type, sampling time, etc. Zhou et al. (2022a) investigated how various soil conditions affected the physiology of soybeans throughout their flowering stage in response to drought stress. When there was enough water available, soybean performance in chernozem, albic soil, and black soil was nearly identical, but there were differences under drought stress, especially the soybean growing in black soil showed the strongest drought resistance. In addition, the use of soil drought for root research is actually very unfriendly. Drought may cause soil to agglomerate, and it is bound to cause different degrees of damage when obtaining roots, which also leads to large errors in root phenotypic data. Zhang et al. (2022a) used 30% sandy loam and 70% fine sand as experimental soil in the study of soil drought on the root morphology of ryegrass and brome, which could reduce the damage of root sampling to a certain extent, but, at the same time, the difference of soil type was changed. Some scholars have proposed to use the combination of vermiculite and perlite to replace the soil to simulate drought, which is indeed an effective measure, but because this soil does not contain nutrients, water control will inevitably lead to the lack of nutrients in the plant, thus interfering with the experimental results (Seminario et al., 2017). In field studies, drought research is limited to irrigation facilities. In a study of wheat, different drought treatments were set up: (1) no irrigation at all from seed to harvest; (2) limited irrigation, with just one irrigation at the jointing stage; and (3) sufficient irrigation, or well irrigation with one irrigation at the jointing stage and proper irrigation at the filling stage (Zhou et al., 2022b). This experiment is usually highly reliable, based on a large number of phenotypic data can reflect the true state of the plant, but the premise is that the natural precipitation between seasons should be relatively stable.

Plants that experience soil drought reveal dehydration of both their cell walls and their cells. Consequently, in order to replicate the dehydration effects of soil dryness, osmotic agents must possess a molecular weight that is large enough and be able to pass through cell walls. The study problem will get more complex if the osmotic pressure passes through the cell wall, enters the cell through the cell membrane, and is metabolized or has other impacts. Common osmotic substances include mannitol, PEG6000 (polyethylene glycol 6000), PEG8000, etc, the drought stress caused by these substances can be collectively referred to as “osmotic stress” (**Figure 1B**) (Noman et al., 2019; Chen et al., 2022a; Luo et al., 2023). Different osmotic substances have different effects. He et al. (2019) studied the effects of mannitol and PEG6000 on maize seedlings under the same osmotic potential, and found that PEG6000 as an osmotic agent was less harmful to plants than mannitol. At present, PEG is still the most widely used substance in the experiment. The molecular formula of PEG, an ethanol polymer, is HOCH_2_-[CH_2_-O-CH_2_]^n^-CH_2_OH. There are 200–20,000 molecular weights in the range. PEG is a perfect regulator of water potential for imitating soil dryness since molecules with a molecular weight of 6000 or higher cannot pass through cell walls (Zhang et al., 2004). The biggest advantage of using PEG is that it can accurately control the water potential and reflect the state of the plant under a specific osmotic potential (Guan et al., 2022). In the experiment, PEG can be used in combination with nutrient solution to measure the phenotypic changes of plants under hydroponic conditions, or PEG and nutrient solution combined with sand culture to simulate drought stress for a long time (Zhou et al., 2022; Wei et al., 2023); many plant drought-responsive signature metabolites, proteins, or genes have been studied based on PEG models (Li et al., 2020; Cao et al., 2022; Yao et al., 2022). However, the biggest controversy at present is that PEG is essentially an osmotic stress that can quickly cause water loss in plants. In fact, the decrease of soil water content during natural drought is a slow process, so whether PEG and soil drought can be collectively referred to as drought stress remains to be confirmed. It can usually be called ‘simulated drought’. In addition, in the use of PEG, long-term application may cause salt accumulation in the sand culture environment, and thus produce toxic effects. Therefore, water is usually irrigated every three days to remove excessive accumulation of PEG in the sand (Wang et al., 2022c). Verslues et al. (2006) reported that the nutrient solution containing PEG has the characteristics of high viscosity, which affects the diffusion of oxygen to the roots and may lead to hypoxia (based on medium culture conditions), but combined with sand culture and regular irrigation of water can alleviate this situation to a certain extent. They also proposed the use of PEG injection into agar plates for treatment to simulate drought, but this use condition is usually limited by plant species or organs, such as seeds, germinated seedlings, or Arabidopsis thaliana (**Figure 1C**). PEG, mannitol and other substances belong to osmotic stress, which can lead to oxidative stress in plants, resulting in a large amount of reactive oxygen species (ROS). High sugar (high glucose or sucrose) can also be used to simulate osmotic stress, but due to the particularity of the material may lead to changes in other pathways. Some scholars have found that the antioxidant system of yeast is activated under high glucose conditions and affects the accumulation of trehalose (Shi et al., 2019). In general, we believe that the use of PEG to simulate drought stress is still an effective method to study plant drought response under fixed water potential conditions.

Hydroponic drought method (also known as drought dehydration method) is also a method to study drought stress, but it is not often used. Suspended the leaves in a uniform flow of air for dehydration treatment, dehydrated at a constant temperature for a fixed time and detected the relative water content of the leaves to define the degree of drought stress (Liu et al., 2018), which is simply to regulate the water status of the leaves (Campany et al., 2021; Luo et al., 2022); the main advantage of this method is that the water potential can be accurately controlled by real-time monitoring of the machine, and it has high repeatability. The study of trees can also predict their drought tolerance through leaf hydraulic traits (Oliveira et al., 2022). The disadvantage is that the experiment on the leaves needs to be carried out in the dark, because the reactive oxygen species mediated by the photosynthetic electron transport chain may cause damage to the plants. This method has great limitations on the experimental site and is only allowed to be carried out under indoor fixed experimental equipment (Liu et al., 2018). A model of drought stress on vegetable roots brought on by a hydroponic system was presented by Koyama et al. (2012)(**Figure 1D**). First, the five-day-old seedlings were moved to a hydroponic system where they were irrigated with an aerated nutritional solution and supported with a plastic foam bed. Following 23 days, the water level in the solution bucket was adjusted to subject the seedlings to stress treatment at various stages before harvest. An obvious advantage of this method is that the phenotypic changes of roots can be observed intuitively, and no additional equipment is needed, which may be easier to fine-tune the stress level. Its disadvantage is that long-term root soaking in water may cause root hypoxia, increase the susceptibility to pests and diseases and a series of adverse factors affecting growth.

## 3 Research progress of the study of drought stress response

Drought often occurs at various developmental stages of crops, such as seed germination, seedling growth, flowering, pollination, and seed setting, and is particularly sensitive to water changes (Dietz et al., 2021). Consequently, plants have adapted by developing a comprehensive array of responses at various levels, including morphological, physiological, biochemical, cellular, and molecular mechanisms, to mitigate the adverse effects of drought stress. These responses usually include photosynthesis and gas exchange, plant relative water content, ion absorption and transport, as well as reactive oxygen species (ROS) -related antioxidant systems, osmotic adjustment systems, and hormone regulation. In addition, various drought-related traits, including root traits, leaf traits, osmotic adjustment ability, water potential, ABA content, and cell membrane stability, have been used as indicators for assessing plant drought resistance (Fang and Xiong, 2015; Sallam et al., 2019; Bandurska, 2022). At present, there have been published a number of plant drought research papers, these studies for different aspects (such as plant hormones, signal perception, etc.) were specifically addressed (Zhu, 2016; Qi et al., 2018; Waadt et al., 2022; Wang et al., 2022a; Zhang et al., 2022c). In this section, we reviewed the morphological, physiological and molecular studies of plant drought response.

**Figure 2:**
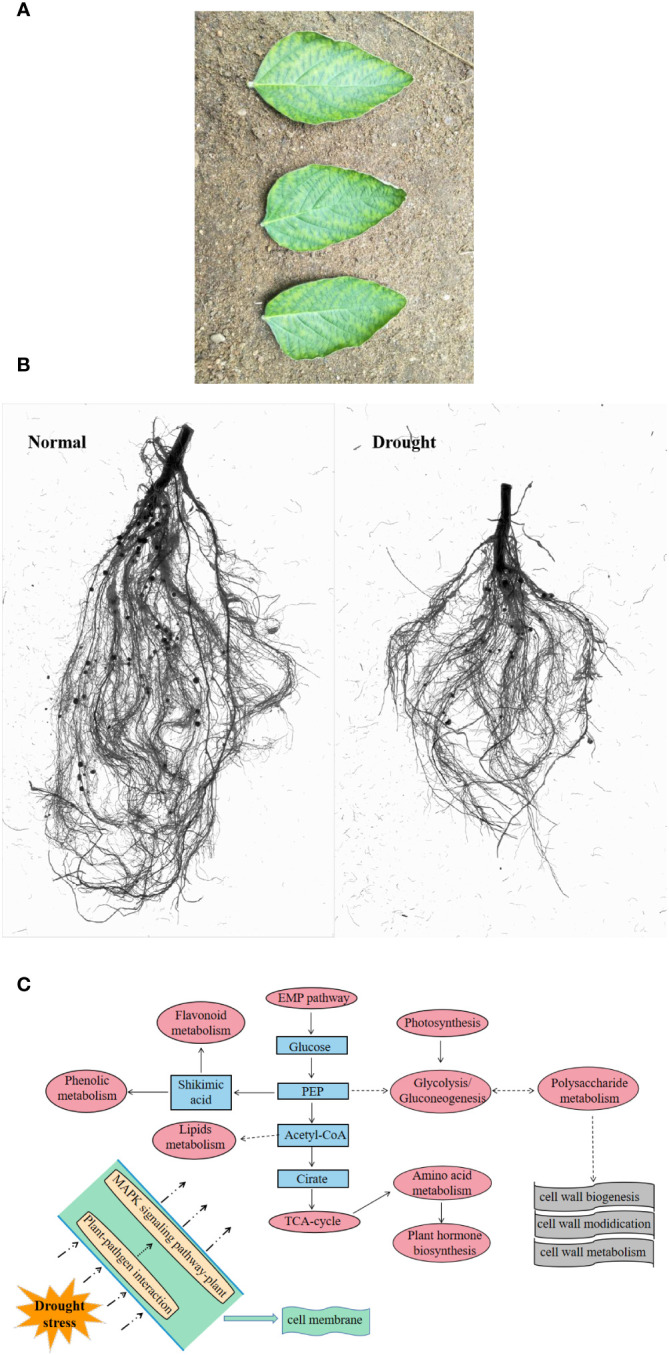
Response of plants under drought stress. **(A)**, leaf state (Wang et al., 2022b); **(B)**, root state; **(C)** preliminary response mechanism of plants (Wang et al., 2022c).

Phenotype can directly reflect the state of plants (De Vienne, 2022). In the above-ground part, drought led to curling and accompanied by partial yellowing of the soybean leaf edge, with signs of water loss (**Figure 2A**) (Wang et al., 2022b); in the underground part, drought causes root development to be blocked (**Figure 2B**). Drought stress also reduced plant height, biomass, leaf area and many other phenotypic traits (Tan et al., 2023). In the underground part, drought led to a decrease in the number of root surface area, root tips, root volume, root activity and root dry weight of rice, thereby inhibiting root growth (Jing et al., 2024). In general, drought-induced long-term changes inhibit the morphological parameters of plants, but short-term stress may be beneficial to plant growth. Within the range of plant self-regulation ability, drought induces an increase in morphological parameters within a certain period of time (Yang et al., 2021).

Drought involves a large number of physiological and biochemical processes, which can be divided into photosynthesis, antioxidant system, osmotic regulation system, membrane lipid peroxidation, hormone signal transduction, ion signal transduction and so on (Bashir et al., 2021). According to Zhang et al. (2022b), flag leaves of wheat with varying drought resistance showed a decrease in the net photosynthetic rate, transpiration rate, stomatal conductance, maximum photochemical efficiency of PSII, and actual photochemical efficiency of PSII (ΦPSII), but the range of the decrease varied from variety to variety. Drought stimulates the physiological response of soybean, such as the single peak change trend of antioxidant enzymes, the accumulation of osmotic adjustment substances, and the increase of MDA content. According to Wang et al. (2022d), T-AOC, antioxidant enzyme activity, osmotic adjustment compounds, and the degree of membrane lipid peroxidation rose most under black soil conditions when the soil moisture content was 15.5%. Plant species and cultivars, as well as the length of time they are exposed to stress, all affect how resistant a plant is to drought. The production and build-up of osmoprotectants, or osmotic pressures, such as soluble proteins, sugars and sugar alcohols, quaternary ammonium compounds, and amino acids, is what controls a plant’s osmotic regulation at low water potential. One of the most crucial amino acids for plants in response to drought is proline (Ozturk et al., 2021). It has powerful functions in maintaining cell homeostasis, regulating plant development and promoting stress adaptation. It is an indispensable and important indicator in drought research (Alvarez et al., 2022). In addition, various plant hormones are induced by drought stress, and ABA is the core to form crosstalk to regulate various processes in response to drought stress (Verma et al., 2016). Ou et al. (2023) reported that drought stress led to the decrease of IAA/ABA, SL/ABA and ACC/ABA values in the aboveground part of tall fescue, indicating that the aboveground part resisted drought by inhibiting growth and delaying organ abscission. The values of IAA/ABA, IAA/GA, IAA/SL and IAA/ACC in the underground part decreased, and the value of SL/ABA increased, indicating that the underground part mainly resisted drought stress by increasing the synthesis of strigolactone. Drought is mediated by abscisic acid to carry out the whole plant perception and signal transmission. Plants produce ABA in a variety of organs in response to stress and start defense processes. Plants are endowed with resistance to environmental stress through the regulation of stomatal aperture and the expression of defense-related genes (Lim et al., 2015; Mukarram et al., 2021). Along with being crucial in regulating stomatal closure through the use of abscisic acid and reactive oxygen species, the calcium ion (Ca^2+^) signal is another vital component in drought response. The receptor kinase HPCA1 (HYDROGEN PEROXIDE-INDUCED CA^2+^ INCREASES1) can directly sense the apoplastic H^2^O^2^, which causes the activation of Ca^2+^ channels on the guard cell membrane and an increase in Ca^2+^ in the guard cytoplasm, ultimately resulting in stomatal closure (Ketehouli et al., 2022; Liu et al., 2022). Additionally, exogenous calcium ion therapy can increase antioxidant enzyme activity, which can somewhat increase plant resilience to drought (Xu et al., 2019).

Under drought stress, plants also remove excessive ROS by accumulating biochemical substances to alleviate oxidative stress. Plants resist drought stress by developing various biochemical, structural and molecular strategies, including the accumulation of certain osmolytes, such as proline, protein, sugar and glycine betaine (Wahab et al., 2022). Under water limitation, H_2_O_2_ (hydrogen peroxide), total soluble protein, glycine betaine, AsA (ascorbic acid) and total phenols are usually accumulated in plants (Kosar et al., 2021). These indicators have been considered as one of the characteristics of plant drought resistance, and genomic loci for proline and hydrogen peroxide accumulation have also been identified (Kamruzzaman et al., 2022). In addition to this, there is glutathione (GSH), which removes ROS through AsA-GSH cycle, GRX (glutaredoxin), GST (glutathione S-transferase) and other enzymatic reactions (Gill et al., 2013). In recent years, more and more studies have confirmed the important role of phenylpropanoid and flavonoid metabolites in drought response, such as the significant increase of afrormosin-7-O-(6’’-malonyl) glucoside (iso1) and glycitein under drought stress. On the other hand, the terminal oxidase POD of the phenylpropanoid pathway is related to the synthesis of lignin (Wang et al., 2024). The accumulation of lignin in leaves and roots is conducive to enhancing the drought tolerance of plants, and some regulatory genes for lignin synthesis have also been identified (Jung et al., 2022). In summary, with the continuous updating of technical means, more and more drought-resistant biochemical reactions have been discovered, and these findings will be further used to assist drought-resistant breeding.

The intricate process of plant drought tolerance is too complex to be entirely explained by changes at the physiological level. Specific genes or small molecule metabolites typically mediate the creation of specific enzymes or hormones. With the deepening of research, the molecular mechanisms of various drought responses have been continuously revealed. Some genes or metabolites have been shown to have the effect on regulating drought. Common metabolites, like isoflavones, have potent antioxidant qualities and can shield DNA from damage caused by free radicals; two significant and potent antioxidants found in soybean isoflavones are genistein and daidzein (Shen et al., 2022). There are also various amino acids that can be degraded to provide ATP sources for the TCA cycle of plants, which can help improve drought stress tolerance (Karami et al., 2023). For example, proline is often used as an penetrant, which can regulate redox balance and energy state (Zheng et al., 2021); the serine family plays a role in sugarcane response to drought stress (Diniz et al., 2020). There are also some phenolic acid metabolites that scavenge reactive oxygen species by activating the phenylpropanoid pathway (Sharma et al., 2019; Köhler et al., 2020). It has also been demonstrated that a few transcription factors, such as AREB/ABF, DREBs, AP2/ERF, bZIP, NAC and MYB, are the primary players in water stress signals, which react to drought by altering stomatal movement or certain metabolic pathways (Lata and Prasad, 2011; Baldoni et al., 2015; Joshi et al., 2016). It has also been discovered that drought-treated tobacco leaves exhibit either up- or down-regulated levels of heat shock protein, thioredoxin, ascorbic acid, glutathione, and hydrogen peroxide-related proteins (Xie et al., 2016). Here, based on the omics data, taking soybean as an example, we summarized the preliminary response pattern of cellular response to drought. Sensors on the cell membrane, such as cell surface pattern recognition receptors (PRRs), activate the MAPK signaling pathway in response to drought stress, activating the defense genes of antimicrobial substances. Furthermore, the synthesis and modification of cell walls occurs outside the cell membrane as a result of the activation of drought resistance pathways based on the TCA cycle, EMP pathway, and glycolytic process (in terms of transcription and metabolism), which includes photosynthesis, hormone metabolism, amino acid synthesis, phenol and flavonoid metabolism, and lipid metabolism (**Figure 2C**). Different varieties may also have distinct mechanisms for drought resistance, such as sulfur, vitamin B6, butyrate metabolism, etc., depending on how they differ from one another (Wang et al., 2022c).

In terms of genetic breeding, researchers have also achieved some gratifying results. The construction of transgenic plants overexpressing MYB14 in soybean not only produces a semi-dwarf and compact plant structure, but also increases yield and drought resistance under high-density planting conditions in the field (Chen et al., 2021). Overexpression of some transcription factors such as GmMYB84 also contributes to drought resistance in soybean (Wang et al., 2017). By coordinating the expression of a set of genes related to drought stress and the nitrate transporter NRT2.5, Zhou et al. (2022) found that overexpression of *GmTDN1* enhanced the photosynthetic and osmotic adjustment ability, antioxidant metabolism, and root quality of wheat plants. This was achieved through heterologous expression of the soybean *TDN1* gene in wheat. In other words, transgenic wheat containing *GmTDN1* has improved drought tolerance and nitrogen uptake. Grain yield increased, membrane damage decreased, osmotic adjustment and photosynthetic efficiency were enhanced, according to another study that also discovered that overexpression of *GmDREB1* from soybean in wheat varieties, transgenic plants grown under restricted water conditions in the field, yield performance and a variety of physiological traits were significantly improved (Zhou et al., 2020). Due to the complexity of drought stress and the continuous innovation of technology, the research on drought stress will continue, and more deep-seated mechanisms will be further revealed.

## 4 Methods to improve drought tolerance

In production, researchers have improved the drought resistance of plants in many ways. In this section, the main methods to improve the drought resistance of crops are summarized.

### 4.1 Plant-microbe interaction

Plant drought stress is significantly reduced by plant growth-promoting rhizobacteria (PGPR). PGPR has a wide range of functions. It can not only ensure the survival of plants during drought, but also promote the growth of plants through various mechanisms such as osmotic adjustment, increased antioxidant activity, and plant hormone production (Sati et al., 2023a). By producing extracellular polysaccharides (EPS), plant hormones, and 1-aminocyclopropane-1-carboxylate (ACC) deaminase, these advantageous microbes infiltrate the rhizosphere and inner rhizosphere of plants and give them drought tolerance. In addition, PGPR can also produce volatile compounds, regulate osmotic pressure, accumulate antioxidants, and up-regulate or down-regulate the expression of stress-responsive genes; at the same time, PGPR can also change root morphology and further enhance the drought tolerance of plants (Vurukonda et al., 2016; Zhang et al., 2021). With the deepening of research, more functions of PGPR have been reported, including nitrogen fixation, phosphate solubilization, siderophore and extracellular polysaccharide production, enhancing root and shoot systems, increasing photosynthetic rate and carotenoid content (Ahluwalia et al., 2021). Some strains, such as Bacillus and Pseudomonas, have high levels of proline, protein, IAA and GA. Under drought stress, plants are endowed with certain drought tolerance through interaction with roots (Carlson et al., 2020; Umapathi et al., 2022). Other researchers have reported that some drought-tolerant PGPR strains are beneficial to plants, which can effectively mobilize nutrients and improve plant material accumulation under drought conditions (Sati et al., 2023).

### 4.2 Genetic engineering for crop improvement

Targeted genome editing has only been accomplished thus far using three methods: zinc finger nuclease (ZFN), transcription activator-like effector nuclease (TALEN), and clustered regularly interspaced short palindromic repeat-Cas9 nuclease (CRISPR-Cas9) (Gupta et al., 2019). Among different genome editing methods, CRISPR-Cas9 has a wider applicability to crop plants and has been used for crop improvement, especially in drought tolerance, yield and domestication (Bhat et al., 2021; Sami et al., 2021). Liu et al. (2021) designed quantitative variations of maize yield-related traits by using CRISPR-Cas9 genome editing to produce weak promoter alleles of the CLE gene and newly identified partially redundant compensated null alleles of the CLE gene, supporting the great potential of genome editing in crop improvement. In addition, there is transgenic technology, which regulates the drought tolerance of crops by promoting the overexpression or heterologous expression of a gene. The specific case has been introduced in the literature (Wang et al., 2017; Zhou et al., 2020; Chen et al., 2021; Zhou et al., 2022). Through molecular breeding or transgenic methods, it provides significant phenotypic and genetic information for the production of drought-tolerant crop varieties (Arya et al., 2021). In summary, multiplex CRISPR/Cas9 should be mentioned as a useful tool to study protein function in crop plants with large polyploid genomes and large families of homoeologous genes.

### 4.3 Chemical regulation to enhance drought resistance

**Figure 3:**
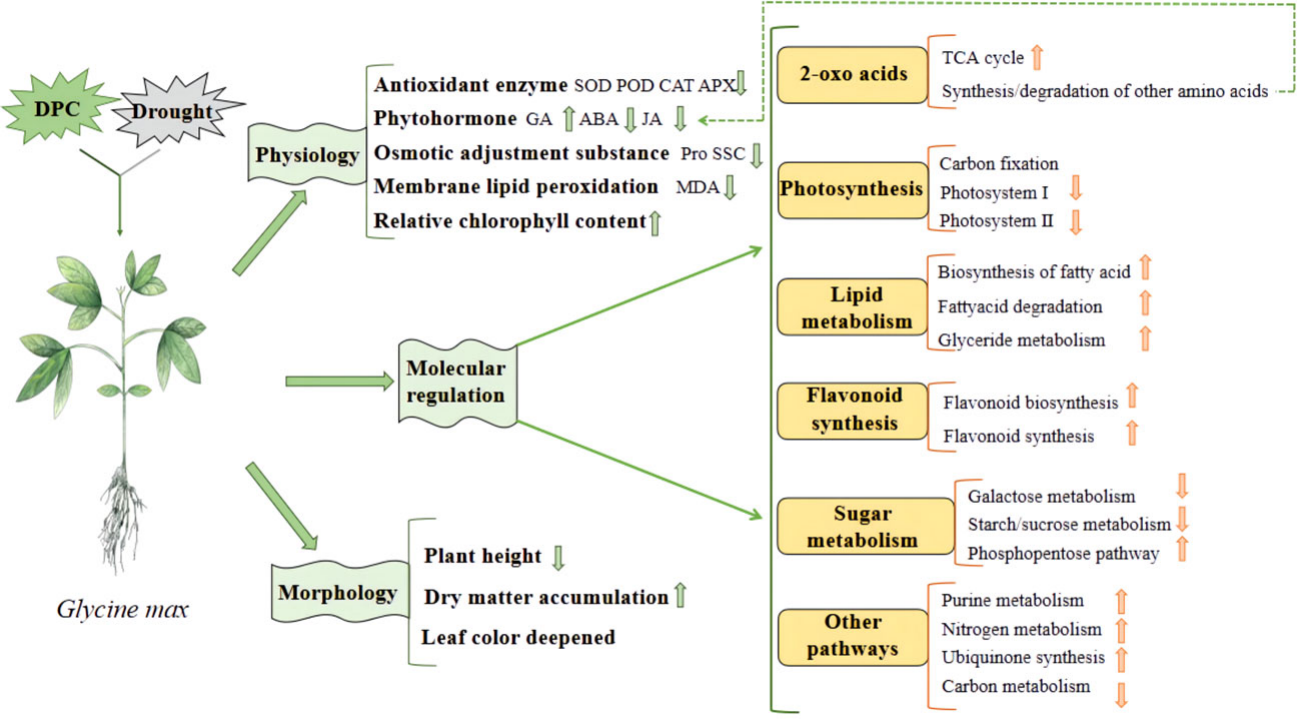
The mechanism of mepiquat chloride regulating soybean response to drought (Wang et al., 2023a).

Chemical regulation is the most commonly used method in production. Commonly used chemical regulators include various plant hormones and their derivatives, plant growth regulators, etc. For example, indole butyric acid, abscisic acid, gibberellin, melatonin, salicylic acid, 6-benzylaminopurine (6-BA) and brassinolide, etc. These exogenous hormones can usually promote seed germination, seedling growth and development, increase the content of osmotic adjustment substances in leaves, antioxidant enzyme activity and chlorophyll content, reduce the relative conductivity and malondialdehyde content of seedling leaves, thereby improving the drought resistance of crops (Arnao and Hernández-Ruiz, 2018; Ahmad et al., 2023; Liu et al., 2023). Some plant growth regulators, such as mepiquat chloride, are commonly used to regulate cotton plant type, but can also increase soluble protein content and antioxidant enzyme activity (Zhang et al., 2022d). It can also promote the accumulation of flavonoids in soybeans to enhance drought resistance (**Figure 3**) (Wang et al., 2023a). There are also some other regulators, such as 24-epibrassinolide·triacontanol, indolebutyric acid·triacontanol, indolebutyric acid·S-abscisic acid, kinetin and triacontanol·6-benzylaminopurine, which have similar ways to improve drought resistance (Lv et al., 2023).

### 4.4 Emerging nanomaterials for enhanced drought resistance

The application of nanotechnology is to respond to climate change-mediated environmental stressors through nanomaterials (such as pesticides, nanobiosensors, nanoclays, and nanoseed priming techniques). In recent years, some nanomaterials have played a significant role in the improvement of crop agronomic traits. Functional carbon nanodots (FCNs) have positive effects on many physiological processes in plant vegetative and reproductive growth stages, including photosynthesis, antioxidant system, osmotic regulation, soil physical and chemical properties and microbial environment improvement (Chen et al., 2022b). Hatami et al. (2017) reported that low concentrations of single-walled carbon nanotubes (SWCNT) treatment can induce plant tolerance to low-to-moderate drought by enhancing water uptake and activating plant defense systems. Some metal nanomaterials have also been used to cope with drought stress. Zinc oxide nanoparticles (ZnONPs) promoted the increase of plant height, total chlorophyll content, plant fresh weight and dry weight, seed and straw yield of rice under drought, reduced MDA content, increased proline level and antioxidant enzyme activity (Waqas Mazhar et al., 2022). Calcium oxide nanoparticles (CaO_NPs) maintain the redox state of *Trachyspermum ammi* L. Sprague by regulating non-enzymatic antioxidants and enzymatic antioxidants, and significantly improve the morphological-agronomic traits of plants (Mazhar et al., 2023). Nanomaterials have great potential in the application of plants.

## 5 New methods and techniques for studying plant adversity

With the continuous progress of science and technology, some new methods and new technologies are emerging. This section introduces some emerging technologies in recent years for plant stress research.

### 5.1 Crop phenomics and high-throughput phenotypic analysis

**Figure 4:**
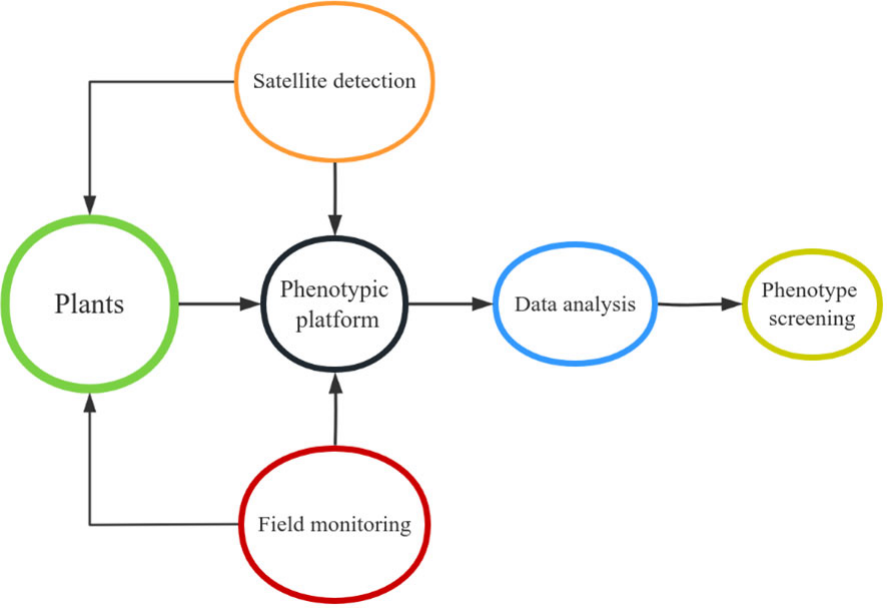
Operation mode of plant phenotype platform.

With the completion of genome-wide sequencing of many crop species and the rapid development of high-throughput phenotypic technology, the genetic information and functional characteristics of plants have been further identified, which has laid a suitable foundation for advanced precision agriculture and improved genetic benefits (Li et al., 2022). However, one of the primary obstacles impeding crop breeding and functional genomics research is the gathering of large-scale phenotypic data. We can now investigate novel approaches for large-scale phenotypic data collection and processing in the upcoming years in order to potentially relieve this bottleneck, thanks to recent technical advancements (Yang et al., 2020). Crop phenomics and high-throughput phenotypic analysis techniques can accurately measure the required traits of thousands of field-grown plants in diverse environments (**Figure 4**). This is a key step in selecting lines with better yield, disease resistance and stress resistance to accelerate crop improvement programs. It helps to reveal the genetic basis of complex traits and targeted traits related to plant growth and development, and provides more in-depth insights and more effective strategies for crop improvement (Jangra et al., 2021). Precise high-throughput phenotypic analysis will hasten genetic advancement of plants and foster the upcoming green revolution in agricultural breeding.

### 5.2 Drought monitoring model based on machine learning

Due to its superior performance in prediction tasks, machine learning (ML) is widely applied in scientific research. Nonetheless, in real-world scenarios like catastrophe tracking and evaluation, the consequences of model malfunction, particularly incorrect negative forecasting, could greatly affect the community. Consequently, stakeholders can improve their understanding of the multifaceted effects of the drought at the regional level and implement suitable countermeasures by utilizing interpretable machine learning to highlight the possibility of broad social benefits (Zhang et al., 2023). A machine learning system called DroughtCast was created by Brust et al. (2021) to forecast US drought monitoring (USDM). In order to accurately estimate USDM over the next one to twelve weeks, DroughtCast uses satellite-observed soil moisture and simulated weather as inputs to a recurrent neural network. A supervised machine learning framework that supplements the rice gene regulation and association network (GRaiN) was proposed by Gupta et al. (2021). The role of OsbHLH148 transcription factors is predicted with accuracy by this approach. In addition to aiding in the genetic engineering of optimal rice varieties, this network and complementing machine learning techniques anticipate important regulatory genes underlying other agricultural features. In addition, hyperspectral images of drought phenotypes, combined with relevant physiological indicators for modeling to predict the drought tolerance of plants, can also be used as a new screening method for evaluating drought-tolerant germplasm resources (Chen et al., 2022c).

### 5.3 The rise of single cell sequencing and spatial omics

In order to fully and deeply understand the function of organisms, we need to conduct in-depth research on their basic unit ‘cells’ in their natural space environment. Single cell sequencing technology provides us with a method to study intracellular dynamics at the single cell level and to answer biological questions through high-dimensional data sets of millions of cells (Mo and Jiao, 2022). Single-cell transcriptomics provides an in-depth understanding of single-cell transcriptomes, while spatial transcriptomics helps to preserve information on spatial relationships between cells. In recent years, these techniques have made significant progress. Giacomello (2021) reviewed modern advances in single-cell RNA sequencing and outlined techniques for moving the plant field into spatial transcriptomics, while describing available spatial transcriptomics methods and specific application examples. However, there are certain drawbacks to these technologies as well, such as their limited applicability, lack of spatial information, or poor resolution; single-cell transcriptomics, spatial transcriptomics, and spatial element distribution together can offer more fruitful avenues for plant research (Chen et al., 2023).

Without requiring any chemical modification or labeling, mass spectrometry imaging (MSI) is a potent method that may directly describe the chemical characteristics and spatial distribution of various substances (Dong and Aharoni, 2022). It maps certain molecules to specific tissue distribution of the original sample, integrates quantitative and qualitative molecular information with spatial information, and enhances conventional chemical analysis (Dong et al., 2016). Simultaneously, the integration of MSI with additional analytical methods has significantly broadened the comprehension of sample data, which is essential for clarifying the processes of endogenous drug synthesis, accumulation, and control (Dong and Aharoni, 2022). For example, Xia et al. (2023) employed a combination of mass spectrometry imaging techniques and metabolomics to examine the spatial distribution of diterpenoids in two types of Salvia miltiorrhiza. They also identified the precise tissue distribution and mechanism of diterpenoids in the leaves, phloem and xylem, root periderm, and root epidermis.

Understanding the potential heterogeneity of complex biological systems is aided by single-cell RNA sequencing. Protein measuring has also been made feasible by technological advancements, which has helped to clarify the different cell types and states found in complicated tissues. Mass spectrometry has advanced on its own recently, bringing us one step closer to defining the single-cell proteome. It is still difficult to identify proteins in single cells using mass spectrometry and sequencing-based techniques (Bennett et al., 2023). However, single cell proteomics is still in the preliminary research stage and has been constructed in the medical field (Wang et al., 2023). In the future, it may provide a new perspective for plant stress research.

## 6 Outlook

With the deepening of research on various types of plants, many drought resistance mechanisms have been revealed. In the current research, there are still some problems to be solved. First, due to the rapid development of high-throughput technology, a large number of drought-responsive genes have been discovered, but in fact, the genes that can be used for genetic improvement are very limited, and there are still many known genes that have not been used. In the future research, in addition to the development of new genes, we should also pay attention to the in-depth study of discovered genes. Second, linked to yield, the ultimate goal of drought research is to reduce yield loss. Therefore, in the study of gene function, it is necessary to move from the laboratory to the field, at least to ensure that the yield is not reduced. Thirdly, root research was carried out to evaluate the overall situation of plants and integrate data (based on data integration, the mechanism of root and leaf synergistic response to drought was analyzed). In the existing research, a large number of experiments are based on the ground, and little is known about the root system. As the primary water sensing organ, the role of roots in drought resistance should not be ignored. Zia et al. (2021) reviewed the integrated rhizosphere management strategies for plant drought stress alleviation, including rhizosphere engineering by adding drought-tolerant bacteria, nanoparticles, liquid nanoclay, nutrients, organic matter, and plant modification using next-generation genome editing tools (such as CRISPR/Cas9), providing theoretical support for further deepening root research in the future. Researchers should pay attention to the comprehensive response of aboveground and underground parts, and clarify the overall dynamic changes or transport patterns of plants through data mining and integration. Of course, this work is complex and still requires a lot of time to explore. Finally, innovative thinking is particularly important. The heterologous expression of genes in different species provides us with new ideas and directions. A gene may have unexpected effects in other species, which is worthy of our continuous exploration.

In conclusion, this paper provides drought researchers with specific viewpoints from methodology to consequentialism, and further deepens the understanding of drought stress. These viewpoints provide theoretical support for researchers to design drought-resistant crops and improve drought tolerance.

## Author contributions

XW: Writing – original draft. XL: Writing – review & editing. WZ: Investigation, Writing – original draft. XH: Investigation, Writing – original draft. SD: Writing – review & editing.
